# Supplementary material for: Maturational Changes Alter Effects of Dietary Phytase Supplementation on the Fecal Microbiome in Fattening Pigs
Source: Microorganisms. 2020 Jul 18;8(7):1073. doi: 10.3390/microorganisms8071073 (PMC7409029; doi:10.3390/microorganisms8071073)
Supplement: Supplementary file 1 [file microorganisms-08-01073-s001.pdf]

Supplementary Table 1. Relative abundance of bacterial phyla in pigs over time.

Phylum	Week				SEM	*P*-value
	**0**	**3**	**5**	**10**		
Firmicutes	60.20^{b}	64.12^{a}	63.44^{a}	63.87^{a}	0.903	0.004
Bacteroidetes	31.68^{a}	28.14^{b}	27.36^{bc}	25.56^{c}	0.845	<0.001
Spirochaetes	2.62^{b}	2.97^{b}	3.79^{a}	4.13^{a}	0.252	<0.001
Proteobacteria	2.15^{ab}	2.40^{a}	2.30^{a}	1.64^{b}	0.242	0.067
Verrucomicrobia	1.46^{a}	0.67^{c}	1.00^{b}	1.37^{a}	0.121	<0.001
Planctomycetes	0.39^{bc}	0.16^{c}	0.46^{b}	1.64^{a}	0.104	<0.001
Actinobacteria	0.59^{a}	0.58^{a}	0.44^{b}	0.28^{c}	0.038	<0.001
Euryarchaeota	0.16^{c}	0.28^{bc}	0.37^{b}	0.76^{a}	0.057	<0.001
Tenericutes	0.12^{b}	0.11^{b}	0.20^{a}	0.15^{ab}	0.026	0.070
Cyanobacteria	0.19^{a}	0.15^{b}	0.15^{b}	0.069^{c}	0.014	<0.001
WPS-2	0.019^{b}	0.13^{ab}	0.12^{b}	0.25^{a}	0.043	0.003
Lentisphaerae	0.12^{a}	0.071^{b}	0.14^{a}	0.13^{a}	0.016	0.003
TM7	0.064^{b}	0.10^{a}	0.12^{a}	0.092^{ab}	0.0135	0.016
Fibrobacteres	0.073^{a}	0.025^{b}	0.022^{b}	0.028^{b}	0.0091	<0.001
Deferribacteres	0.054^{a}	0.039^{b}	0.036^{b}	0.011^{c}	0.0054	<0.001
Chlamydiae	0.054^{a}	0.032^{b}	0.013^{b}	0.0071^{c}	0.0069	<0.001
Elusimicrobia	0.015^{ab}	0.012^{b}	0.029^{a}	0.011^{b}	0.0054	0.073
Synergistetes	0.025^{a}	0.0084^{b}	0.011^{b}	0.0071^{b}	0.0030	<0.001
Fusobacteria	0.0020^{a}	0^{b}	0^{b}	0^{b}	0.0004	0.017

n = 64 per time point; SEM, standard error of the mean.

a,b Different lowercase letters per time point within a row indicate significant difference ($P < 0.05$).

Supplementary Table 2. Relative abundances (0.25% of all reads) of genera in pigs over time.

Week	0	3	5	10	SEM	*P*-value
Prevotella	20.80[a]	20.15[a]	16.98[b]	12.99[c]	0.994	<0.001
Clostridiaceae	6.84[c]	7.43[c]	9.01[b]	11.66[a]	0.566	<0.001
Ruminococcaceae	6.68[b]	7.22[b]	8.02[a]	8.33[a]	0.328	<0.001
Lactobacillus	4.70[ab]	6.22[ab]	4.55[b]	6.87[a]	0.700	0.035
SMB53	3.59[b]	4.83[b]	4.96[b]	5.05[a]	0.275	<0.001
Oscillospira	3.02[c]	3.79[b]	4.42[a]	4.40[a]	0.205	<0.001
S24-7	3.49[b]	2.83[c]	3.58[b]	4.71[a]	0.218	<0.001
Treponema	2.49[b]	2.97[b]	3.74[a]	4.05[a]	0.251	<0.001
Lachnospiraceae	3.49[a]	2.48[b]	2.94[b]	3.50[a]	0.219	<0.001
Bacteroidales	2.97[b]	2.23[c]	2.96[b]	3.58[a]	0.189	<0.001
Ruminococcus	1.95[c]	3.03[a]	3.21[a]	2.45[b]	0.152	<0.001
Megasphaera	3.54[a]	3.49[a]	2.03[b]	0.91[c]	0.316	<0.001
Streptococcus	1.49[b]	1.79[b]	3.02[a]	3.27[a]	0.304	<0.001
Coprococcus	2.01	1.83	2.04	2.00	0.095	0.280
Clostridiales	1.87	1.74	2.05	2.00	0.121	0.217
Roseburia	2.48[a]	2.05[a]	1.3[b]	0.89[b]	0.195	<0.001
Phascolarctobacterium	1.56[ab]	1.55[ab]	1.73[a]	1.39[b]	0.106	0.047
Blautia	1.63[a]	1.70[a]	1.36[b]	0.94[c]	0.104	<0.001
Veillonellaceae	2.32[a]	1.76[b]	1.09[c]	0.35[d]	0.214	<0.001
[Prevotella]	1.93[a]	1.34[b]	1.23[b]	0.94[c]	0.101	<0.001
RFP12	1.47[a]	0.67[b]	1.00[b]	1.34[a]	0.122	<0.001
Christensenellaceae	0.65[c]	0.62[c]	1.08[b]	1.88[a]	0.152	<0.001
Dialister	1.13[b]	1.60[a]	1.09[b]	0.37[c]	0.157	<0.001
Faecalibacterium	1.28[a]	1.28[a]	0.89[b]	0.48[c]	0.097	<0.001
Succinivibrio	0.95[b]	1.36[a]	0.79[bc]	0.53[c]	0.162	<0.001
CF231	0.81[a]	0.60[b]	0.76[ab]	0.84[a]	0.075	0.054
Clostridium	1.09[a]	0.27[c]	0.57[b]	1.01[a]	0.102	<0.001
[Mogibacteriaceae]	0.77	0.68	0.73	0.76	0.051	0.557
Pirellulaceae	0.39[bc]	0.17[c]	0.47[b]	1.67[a]	0.106	<0.001
Turicibacter	0.51[b]	0.55[b]	0.70[a]	0.80[a]	0.055	<0.001
Anaerovibrio	1.09[a]	0.67[b]	0.43[c]	0.28[c]	0.079	<0.001
Dorea	0.69[a]	0.68[a]	0.61[a]	0.48[b]	0.042	<0.001
Butyricicoccus	0.67[a]	0.62[a]	0.60[a]	0.45[b]	0.036	<0.001
Bulleidia	0.47[c]	0.65[a]	0.55[b]	0.35[d]	0.032	<0.001
[Ruminococcus]	0.44[b]	0.60[a]	0.46[b]	0.30[c]	0.034	<0.001
Sarcina	0.076[b]	0.60[a]	0.55[a]	0.47[a]	0.050	<0.001
Peptostreptococcaceae	0.39[b]	0.54[a]	0.34[b]	0.30[b]	0.029	<0.001
Succinivibrionaceae	0.014[c]	0.25[b]	0.74[a]	0.48[ab]	0.155	0.003
[Eubacterium]	0.45[a]	0.50[a]	0.34[b]	0.18[c]	0.029	<0.001
Parabacteroides	0.28	0.28	0.37	0.3	0.045	0.339
RFN20	0.30[ab]	0.29[b]	0.37[a]	0.27[a]	0.028	0.063
BS11	0.36[a]	0.075[b]	0.20[ab]	0.56[a]	0.093	<0.001
RF16	0.42[a]	0.15[b]	0.25[b]	0.36[ab]	0.055	<0.001
Oribacterium	0.21[b]	0.38[a]	0.35[a]	0.22[b]	0.023	<0.001
Coriobacteriaceae	0.35[a]	0.32[a]	0.27[b]	0.19[c]	0.021	<0.001

Mitsuokella	0.54[a]	0.32[b]	0.22[b]	0.037[c]	0.063	<0.001
Paludibacter	0.27[b]	0.16[c]	0.27[b]	0.40[a]	0.054	<0.001
Methanobrevibacter	0.065[c]	0.15[bc]	0.24[b]	0.60[a]	0.046	<0.001
Campylobacter	0.32[a]	0.29[a]	0.26[ab]	0.17[b]	0.038	0.023
Escherichia	0.37	0.19	0.19	0.24	0.072	0.239

n = 64 per time point; SEM, standard error of the mean.

[a,b] Different lowercase letters per time point within a row indicate significant difference ($P < 0.05$).

Supplementary Table 3. Abundances of bacterial genera in pigs fed diets with or without phytase at the start of the experiment (week 0).

Genus	Mean[1]	$\log_2$ fold change[2]	SE	*P*-value	*q*-value[3]
Prevotella	7900	-0.03	0.226	0.902	1.000
Clostridiaceae	2261	0.01	0.253	0.984	1.000
Ruminococcaceae	2255	-0.01	0.136	0.914	1.000
Lactobacillus	1669	0.70	0.411	0.087	1.000
Megasphaera	1467	0.32	0.479	0.502	1.000
SMB53	1223	0.08	0.197	0.699	1.000
S24-7	1189	-0.02	0.168	0.918	1.000
Lachnospiraceae	1185	-0.16	0.169	0.352	1.000
Roseburia	1038	-0.60	0.391	0.127	1.000
Veillonellaceae	1032	-0.83	0.527	0.116	1.000
Oscillospira	1023	-0.08	0.142	0.582	1.000
Bacteroidales	995	-0.29	0.205	0.163	1.000
Treponema	840	-0.17	0.243	0.471	1.000
Coprococcus	689	0.04	0.111	0.740	1.000
Ruminococcus	687	-0.24	0.147	0.105	1.000
[Prevotella]	654	-0.28	0.145	0.050	1.000
Clostridiales	633	-0.20	0.181	0.271	1.000
Streptococcus	610	0.12	0.455	0.793	1.000
Blautia	590	-0.04	0.206	0.844	1.000
Phascolarctobacterium	527	0.01	0.192	0.969	1.000
Dialister	503	-0.32	0.594	0.589	1.000
RFP12	474	-0.15	0.409	0.721	1.000
Faecalibacterium	472	0.05	0.299	0.858	1.000
Anaerovibrio	399	0.17	0.326	0.599	1.000
Clostridium	349	-0.39	0.411	0.344	1.000
Succinivibrio	339	-0.81	0.425	0.056	1.000
CF231	265	-0.67	0.264	0.011	0.803
[Mogibacteriaceae]	257	-0.25	0.199	0.203	1.000
Mitsuokella	245	-0.58	0.617	0.344	1.000
Dorea	239	-0.15	0.168	0.367	1.000
Butyricicoccus	233	0.27	0.146	0.069	1.000
[Eubacterium]	174	-0.32	0.271	0.232	1.000
Christensenellaceae	172	0.57	0.426	0.181	1.000
Bulleidia	169	0.09	0.181	0.608	1.000
Turicibacter	166	-0.41	0.296	0.162	1.000
Escherichia	161	-0.86	0.955	0.366	1.000
[Ruminococcus]	161	0.07	0.261	0.799	1.000
Acidaminococcus	145	-0.92	1.061	0.387	1.000
RF16	136	-0.14	0.701	0.846	1.000
Peptostreptococcaceae	132	-0.22	0.158	0.171	1.000
Pirellulaceae	131	0.09	0.635	0.891	1.000
Coriobacteriaceae	126	-0.02	0.163	0.908	1.000
BS11	116	-0.77	1.421	0.587	1.000
RFN20	106	-0.15	0.228	0.512	1.000

Campylobacter	103	-0.48	0.301	0.109	1.000
p-75-a5	101	-0.36	0.260	0.164	1.000
Catenibacterium	92	0.30	0.756	0.689	1.000
Parabacteroides	91	-0.09	0.382	0.804	1.000
Paludibacter	90	-0.31	0.733	0.670	1.000
Lachnospira	85	0.10	0.323	0.758	1.000
Collinsella	84	-0.12	0.297	0.687	1.000
Oribacterium	79	0.12	0.377	0.760	1.000
Shuttleworthia	77	1.24	0.762	0.103	1.000
[Paraprevotellaceae]	69	0.21	0.210	0.307	1.000
YS2	69	0.08	0.265	0.769	1.000
Sutterella	64	0.11	0.189	0.569	1.000
Desulfovibrio	57	-0.22	0.224	0.315	1.000

Normalized reads (hit counts). Only the most abundant genera pathways (>0.01% of the mean hit counts) between the two dietary groups are presented. Con, control diet; Phy, phytase diet. Con diet: n = 30; Phy diet: n = 34.

[1] Mean normalized reads across diets.

[2] Standard error of the $\log_2$ fold change.

[3] False discovery rate (Benjamini-Hochberg) corrected *P*-value.

Supplemental Table 4. Bacterial phyla abundances in feces of pigs fed diets with or without phytase supplementation over time.

	Week 0					Week 3					Week 5					Week 10				
**Phylum**	**Mean[1]**	**$\log_2$ fold change[2]**	**SE**	***P*-value**	***q*-value[3]**	**Mean[1]**	**$\log_2$ fold change[2]**	**SE**	***P*-value**	***q*-value[3]**	**Mean[1]**	**$\log_2$ fold change[2]**	**SE**	***P*-value**	***q*-value[3]**	**Mean[1]**	**$\log_2$ fold change[2]**	**SE**	***P*-value**	***q*-value[3]**
Firmicutes	23368	-0.05	0.117	0.701	0.832	14847	0.63	0.153	<0.001	<0.001	13826	0.05	0.146	0.753	0.848	10151	0.15	0.089	0.086	0.154
Bacteroidetes	11954	0.01	0.092	0.946	0.985	5719	-0.15	0.089	0.088	0.133	5543	-0.16	0.115	0.176	0.353	4197	-0.27	0.114	0.020	0.051
Spirochaetes	944	-0.10	0.247	0.678	0.832	579	-0.09	0.195	0.641	0.721	712	-0.41	0.164	0.012	0.037	637	0.12	0.210	0.575	0.690
Proteobacteria	780	-0.42	0.243	0.082	0.452	586	1.01	0.300	0.001	0.002	509	-0.74	0.280	0.008	0.028	250	-0.93	0.266	<0.001	0.003
Verrucomicrobia	516	-0.16	0.405	0.688	0.832	135	-0.29	0.291	0.317	0.439	187	-0.06	0.197	0.752	0.848	217	0.01	0.286	0.969	0.969
Planctomycetes	135	0.14	0.617	0.822	0.919	37	1.08	0.611	0.077	0.125	96	1.27	0.399	0.001	0.009	263	0.92	0.305	0.002	0.009
Actinobacteria	215	0.10	0.151	0.506	0.832	109	0.12	0.252	0.634	0.721	84	0.08	0.232	0.746	0.848	52	-0.81	0.286	0.005	0.014
WPS-2	7	0.62	1.286	0.632	0.832	23	2.60	1.010	0.010	0.026	14	-0.09	0.824	0.917	0.971	42	-1.39	0.668	0.037	0.083
Euryarchaeota	56	0.38	0.223	0.092	0.452	81	2.09	0.383	<0.001	<0.001	85	-0.13	0.331	0.702	0.848	122	1.05	0.310	0.001	0.003
TM7	25	-0.20	0.345	0.564	0.832	29	1.98	0.450	<0.001	<0.001	30	-0.02	0.492	0.974	0.974	15	0.10	0.301	0.741	0.833
Tenericutes	43	0.58	0.459	0.210	0.666	26	2.00	0.447	<0.001	<0.001	38	2.91	0.381	<0.001	<0.001	24	1.58	0.335	<0.001	<0.001
Deferribacteres	21	-0.48	0.455	0.289	0.785	9	1.12	0.525	0.033	0.074	7	1.42	0.501	0.004	0.020	2	1.03	0.830	0.215	0.352
Chlamydiae	21	-0.80	0.477	0.095	0.452	7	0.06	0.655	0.922	0.926	3	0.24	0.703	0.729	0.848	1	0.76	1.023	0.455	0.630
Lentisphaerae	43	-0.01	0.457	0.985	0.985	17	2.06	0.427	<0.001	<0.001	34	2.28	0.392	<0.001	<0.001	20	1.14	0.287	<0.001	0.001
Cyanobacteria	72	0.14	0.269	0.592	0.832	36	0.69	0.364	0.057	0.102	35	-0.70	0.322	0.030	0.078	11	-0.25	0.380	0.508	0.653
Fibrobacteres	27	-1.55	0.567	0.006	0.119	5	-1.53	0.760	0.044	0.088	4	-0.45	0.719	0.533	0.848	4	-1.28	0.685	0.061	0.122
Elusimicrobia	6	-0.69	0.988	0.485	0.832	3	0.68	1.199	0.569	0.721	4	-1.81	0.974	0.063	0.142	2	1.09	0.962	0.257	0.385
Synergistetes	8	-0.88	0.566	0.120	0.455	2	-0.13	1.416	0.926	0.926	2	-0.67	0.934	0.473	0.848	1	0.08	0.972	0.938	0.969
Fusobacteria	1	-1.51	2.218	0.497	0.832	0	NA	NA	NA	NA	0	NA	NA	NA	NA	0	NA	NA	NA	NA

Normalized reads (hit counts). Con, control diet; Phy, phytase diet. Per time point: Con diet: *n* = 30; Phy diet: *n* = 34.

[1] Mean normalized reads across time point.

[2] Standard error of the $\log_2$ fold change.

[3] False discovery rate (Benjamini-Hochberg) corrected *P*-value.

Supplemental Table 5. Alpha diversity metrices for the two fecal 'community type' clusters in pigs.

	**Cluster A**	**Cluster B**	**SEM**	***P*-value**
Chao1	491	764	17.3	<0.001
Shannon	5.043	5.463	0.039	<0.001
Simpson	0.979	0.984	0.001	0.005

Cluster A: n = 231 samples; Cluster B: n = 25 samples; SEM, standard error of the mean.
